# Leveraging the teledermatology platform for local global health: Opportunities (and challenges) for sustainable capacity building

**DOI:** 10.1016/j.jdin.2023.07.007

**Published:** 2023-07-21

**Authors:** Tejas P. Joshi, Danielle Garcia, Rohit Gupta

**Affiliations:** a School of Medicine, Baylor College of Medicine, Houston, Texas; b Department of Dermatology, University of Texas Southwestern Medical Center, Dallas, Texas

**Keywords:** capacity building, global dermatology, Hispanic, Latinx, local global health, Native Americans, rural dermatology, skin neglected tropical diseases, teledermatology, telemedicine, tropical dermatology

*To the Editor:* We read, with interest, the article by Patel et al,^1^ which utilizes a systematic review of studies to explore the impact of teledermatology (TD) interventions on medical students, residents, and global dermatology; we commend the authors for synthesizing the evidence demonstrating the outcomes of TD in this setting. Importantly, the authors systematically analyze the outcomes that TD has among health professionals globally. Here, we wish to add to the discussion initiated by Patel et al^1^ by emphasizing the potential role of TD in expanding dermatologic care to underserved communities within the United States through sustainable capacity building. We also highlight some limitations of TD and offer avenues to overcome these constraints.

During the COVID-19 pandemic, TD was realized as a potential tool to serve local communities with previously limited access to care.^2^ These communities include the Hispanic/Latinx community, the Native American community, and recently resettled refugees. TD can also extend the scope of dermatologic care to rural America, where there is a paucity of board-certified dermatologists. In these settings, TD can be used not only to provide clinical care but also to provide education to primary care physicians. Although more complex skin conditions will ultimately require dermatologist consultation, rural primary care physicians can be trained via TD to better triage common inflammatory and pigmentary lesions, reducing the burden on local dermatologists.^3^ Additionally, as articulated by Patel et al,^1^ TD can also be used to educate medical students and residents, exposing them to conditions with racial or ethnic predilection (eg, erythema dyschromicum perstans, which is more common among Hispanics, or central centrifugal cicatricial alopecia, which preferentially afflicts Blacks).

Certainly, the TD platform is not without its limitations. Although TD can be used to triage patients and manage more straightforward cases, more complex and ambiguous cases will require biopsy. Mobile clinics may be a consideration to offer another way to perform biopsies. For instance, Petrova et al^4^ recently demonstrated the utility of the SmartPod mobile clinic in delivering COVID-19 vaccinations in an underserved area in Houston, Texas. Moreover, suboptimal image quality (in store-and-forward TD) impedes the rendition of accurate diagnoses. In such cases, artificial intelligence applications could serve as a tool to screen for image quality. However, given the lack of diversity in training sets used to train machine learning algorithms, artificial intelligence should be used with caution, lest we propagate existing disparities. Furthermore, although TD can be used to render diagnoses, lack of access to affordable medications remains a serious concern, both locally and globally. Efforts to increase supply of cost-effective therapies that can blunt the burden of dermatologic conditions, with significant loss of disability-adjusted life years (eg, lymphatic filariasis, onchocerciasis, scabies, and yaws), should be simultaneously endorsed.^5^

Altogether, we emphasize that TD be viewed as a tool to build capacity for diverse local communities (local global health). We should make a concerted effort to address the limitations of the TD platform through mobile clinics, artificial intelligence, and investment in efficacious and economic drug delivery programs.

## Conflicts of interest

None disclosed.
